# Stakeholder engagement in the development of genetically modified mosquitoes for malaria control in West Africa: lessons learned from 10 years of Target Malaria’s work in Mali

**DOI:** 10.3389/fbioe.2023.1286694

**Published:** 2024-01-05

**Authors:** Bakara Dicko, Souleymane Kodio, Hatouma Samoura, Fatoumata Traoré, Naima Sykes, Mouhamed Drabo, Delphine Thizy, Isabelle Coche, Benjamin Robinson, Kadiatou Sanogo, Bilkissou Yagouré, Samba Diop, Mamadou B. Coulibaly

**Affiliations:** ^1^ Malaria Research and Training Center at the University of Sciences, Techniques and Technologies of Bamako, Bamako, Mali; ^2^ Imperial College London, London, United Kingdom; ^3^ Emerging Ag, Inc., Calgary, Canada

**Keywords:** stakeholder engagement, gene drive, genetic modification, malaria, Mali, Africa, community engagement

## Abstract

From 2012 to 2023, the Malaria Research and Training Center (MRTC), based out of the University of Sciences, Techniques and Technologies of Bamako (USTTB), was part of the Target Malaria research consortium working towards developing novel gene drive-based tools for controlling populations of malaria vector mosquitoes. As part of this work, Target Malaria Mali has undertaken a range of in-depth engagement activities with the communities where their research is conducted and with other stakeholders nationally. These activities were meant to ensure that the project’s activities took place with the agreement of those communities, and that those communities were able to play a role in shaping the project’s approach to ensure that its eventual outcomes were in line with their needs and concerns. This paper aims to conduct a critical assessment of those 10 years of stakeholder engagement in order to identify good practices which can inform future engagement work on gene drive research in West Africa. It sets out a range of approaches and practices that enabled the Target Malaria Mali team to engage a variety of stakeholders, to share information, collect feedback, and determine community agreement, in a manner that was inclusive, effective, and culturally appropriate. These can be useful tools for those working on gene drive research and other area-wide vector control methods in West African contexts to ensure that their research is aligned with the interests of the communities who are intended to be its ultimate beneficiaries, and to allow those communities to play a meaningful role in the research process.

## Background

The most recent WHO World Malaria Report noted the persistent trend in the rise of malaria cases, which reached 247 million in 2021, up from 211 million in 2015. This represents a dramatic increase in malaria cases, reversing decades of progress in the fight against this disease (World Health Organization, 2017b; World Health Organization, 2021b). Africa shoulders the worst of the malaria burden. In 2021, Africa accounted for about 95% of cases and 96% of deaths globally; with children under 5 years old representing 78.9% of all deaths in the region (World Health Organization, 2021b).

While the causes for this increase are many and complex, it is now broadly acknowledged that new tools and interventions will be needed to complement existing ones to be able to achieve malaria elimination. Target Malaria is one of several projects seeking to address this need for innovative tools by developing new, cost-effective, and sustainable genetic technologies to modify mosquitoes and reduce malaria transmission (Target Malaria, 2017).

As an international not-for-profit research consortium, Target Malaria brings together several partner institutions that collaborate in the development of gene drive mosquitoes for malaria control. Since its start in 2012, it has sought to partner with leading public health research centers in the countries most affected by this disease in Africa. In Mali, this took the form of an in-depth collaborative relationship with the Malaria Research and Training Center (MRTC), based out of the University of Sciences, Techniques, and Technologies of Bamako (USTTB) which was initially formalized in 2012. The team at MRTC formed what is referred to in this article as the Target Malaria Mali team.

During the past decade, as part of Target Malaria, Target Malaria Mali has worked in several sites to conduct entomological studies to inform gene drive research. These activities have ranged from macro-invertebrates and larval collections to swarming and indoor collections. In addition, Target Malaria Mali has renovated its insectary facility and laboratory to meet Arthropod Containment Level 2 standards (Achee et al., 2022). This allowed the Target Malaria Mali team to not only conduct entomological studies but also to import and study non-gene drive genetically modified sterile male mosquitoes in containment.

While no studies involving gene drive modified mosquitoes took place in Mali during the 10-year period described in this article, the Target Malaria Mali team undertook a number of studies that contributed to overall research progress for the project. For all studies, both in field sites for entomology and at the insectary, Target Malaria Mali worked closely with the local communities to share information, involve them in the research, and seek their agreement for various activities. In addition, the team also engaged a wider range of stakeholders at the regional and national level, from district health authorities to the relevant national ministries, to ensure these stakeholders were aware of and understood the work taking place, and to receive their feedback. The early engagement of stakeholders and communities reflects Target Malaria’s commitment to starting outreach and dialogue well before any gene drive mosquito would be available to study in Mali or elsewhere in Africa (Roberts and Delphine, 2022).

As a result of the broad scope of stakeholder engagement efforts undertaken by the team in Mali, the situation, contexts, and main characteristics of different stakeholder groups varied widely. While the local communities where field entomology activities took place are rural, with small populations that are relatively homogenous, stakeholders around the research center were living in an urban environment, with more heterogenous populations. From local community to government-level stakeholders, levels of literacy, preferences for different media (audio/radio, written/papers, etc.) and modes of communication also varied greatly, as well as their preferred language for interaction.

After 10 years of collaboration as part of Target Malaria, the team at MRTC is now spinning off to build a center of excellence in molecular biology and genetics for vector control. The team is building on the knowledge and skills acquired through its experience and expects to continue to collaborate with Target Malaria and other similar projects in the future. The scientific and technical legacy of Target Malaria Mali are evident in the new project, but the 10 years of work have also created a significant body of knowledge and experience in stakeholder engagement that can help inform the growing field of genetic vector control research.

Target Malaria Mali benefited from existing guidance to help shape its activities, such as those developed by America’s National Academies of Sciences Engineering and Medicine (National Academies of Science, Engineering and Medicine, 2016) and by the World Health Organization (James et al., 2018; World Health Organization, 2021a). However, like other teams in the project, it also had to contend with how to bring high-level guidance into practice, in a field that is subject to intense scrutiny due to the novelty that gene drive approaches represent in the field of malaria research and more broadly vector control (Pare Toe et al., 2022).

While community engagement in malaria research is not new and is becoming established as a best practice as well as often a requirement of institutional research committees (World Health Organization, 2020; Pell, 2023), the experience of Target Malaria Mali is unique in several ways: 1) the novelty of the technology being developed; 2) the challenge in putting Target Malaria’s commitment to co-development into practice (Target Malaria, 2017); 3) the need to manage changes in the project given its long timeframe, and eventually the end of the project itself. This paper looks at lessons learned from 10 years of stakeholder engagement, with the hope to contribute to the growing body of knowledge and practice in this field. The growing body of publications on engagement for gene drive research informs the results presented in this paper, but it represents a unique contribution based on the unusual scale and duration of Target Malaria Mali’s engagement.

## Definitions and terminology

With regards to the classification of different groups of stakeholders who may be the subject of engagement activities, this paper uses the following definitions, adapted from suggestions by the National Academies of Sciences, Engineering, and Medicine in the context of gene drive research (National Academies of Science, Engineering and Medicine, 2016), and used in other Target Malaria publications (Pare Toe et al., 2022):

### Communities

Groups of people who live within the geographical location or biologically relevant proximity (e.g., flight distance of a targeted insect vector) to a potential site where research is taking place or where field releases may take place such that they have tangible and immediate interests in the research project. They are included within the broader category “stakeholders”.

### Stakeholders

Organisations, groups, or persons with professional or personal interests sufficient to justify engagement but who may or may not have geographic proximity to potential work or intervention sites of the project.

### Publics

Groups who lack the direct connection to a project that stakeholders and communities have but that nonetheless have interests, concerns, hopes, fears, and values that can contribute and influence decision-making about the research and its potential products.

Target Malaria and its partners are committed to the principle of co-development. In the context of their work, **co-development** has been defined as “a collaborative process of jointly designing a research pathway and its resultant intervention to reach a common goal” (Thizy et al., 2019).

## Methodology

The engagement work carried out by Target Malaria team followed different methodologies that were adapted according to the type of stakeholders engaged. This work was covered by two different types of ethics protocols. Firstly, the protocols for field entomology, which included the methodology for seeking and recording individual consent or community acceptance according to the type of collection method (individual consent for methods such as pyrethroid spray catch that required entering into someone’s room, community acceptance for activities like swarming catch that take place in the common village area). Secondly, the stakeholder engagement protocols that would cover all engagement activities that were not directly related to field entomology. Both type of protocols were reviewed by the Ethics Committee of the Faculty of Medicine, Pharmacy and Odontostomatology. The ethics committee also came on an annual basis to visit some of the communities where the project was operating. During these visits, the committee would often observe an engagement activity–for instance the annual feedback to communities on entomological data collection–and have direct interactions with community members.

Engagement methodologies evolved according to the type of stakeholders and the engagement objectives. For policymakers and authorities, one-on-one meetings were often chosen, in order to provide an appropriate space for them to share their perspective. Those meetings could take the form of a Target Malaria presentation followed by a discussion to collect policymakers’ perspectives, or to respond to any clarification questions. For academics or members of the malaria control programs, the format was more often a small group engagement, usually starting by a scientific presentation about a particular topic related to the research–either prompted by the stakeholders’ requests or proposed by the project–followed by an extensive Q&A session and elicitation of perspectives.

The methodologies employed with local community members were different, and diverse. They often resulted from discussions with community representatives about their preferences, to which the team adapted. Target Malaria Mali used large “town-hall style” meetings where the project would present on the project or a particular component or activity, followed by a traditional oral deliberation process by which various members of the community provide their perspective, which are repeated by other members who add to it, and summarized by a key community figure who concludes on behalf of the community. To complement these community-wide meetings, the project was also using focus groups discussion on specific topics to elicit community members’ perspectives, in particular for more vulnerable groups like women or youth, as well as individual meetings to reach out to specific individuals who might not be available or willing to attend a broader group discussion. The topics of discussion were adapted for each one of these meetings, taking into account the feedback and inputs provided in the previous meetings. For engagement aiming at seeking and recording consent or community agreement for field entomology activities, the methodology was similar to what is described above but different methods were used to explain the consent forms. These built on previous experience with genomic studies, malaria vaccine studies and other entomological studies (Diallo et al., 2005; World Health Organisation, 2014; Traore et al., 2015). The paper will present more in details some of these approaches, which were often driven by stakeholders’ requests or based on evaluations of previous methods.

The project questioned whether its engagement work would require individual consent for each engagement activity. This discussion was held with ethics committees of various African research institutions, as well as with Imperial College London’s Ethics Review Board, and the recommendation was that unless the project was doing a specific study, involving for instance a questionnaire, a written informed consent was not necessary for the general engagement activities. In those cases, the project should explain to stakeholders that they were free to participate or not in those activities and that this would not have an impact on their ability to receive the potential benefits that the project might generate in the future (in terms of a new tool for malaria control). A survey requiring individual consent was done in 2015 to analyze communities’ knowledge of malaria and a second one was carried out to evaluate the usefulness of the videos used in 2019 to explain entomological methods prior to seeking consent for these activities.

The findings presented in this paper are the result of a multi-stage process of discussion and reflection, drawing on the experiences of a cross-disciplinary selection of experts involved in Target Malaria’s work in Mali, including both researchers from MRTC themselves, as well as those tasked with coordinating Target Malaria’s stakeholder engagement work more broadly. The initial stage involved collecting written inputs from these experts, on the basis of which structured oral interviews were conducted to gather further details. The information collected was analysed to determine and refine common elements and thematic clusters of best practices, and to identify illustrative case studies for each of these. The results were circulated several times to all participants to ensure all information was accurate and aligned with their experiences.

## Challenges for community and stakeholder engagement in global health research

The guidance documents for responsible gene drive research are all very clear: community and stakeholder engagement are critical components of this research. For example, the Principles for Gene Drive Research highlight that engagement is “critical for enabling well-informed public discussion and debate that is free from the type of sensational hype that has framed new technology in the past” (Emerson et al., 2017). This paper calls for “meaningful”, “robust, inclusive, and culturally appropriate engagement”. The WHO Guidance Framework for Testing Genetically Modified Mosquitoes considers engagement as “an ethical obligation” playing an “essential role in demonstrating respect for affected communities and fulfilling ethical responsibilities to them” (World Health Organization, 2021a). However, those documents do not provide guidance to researchers on how to practically address key engagement challenges. For instance, on the well-documented issue of stakeholder fatigue in public health research (Clark, 2008), the WHO Guidance only stresses that “consideration must be given to mechanisms to monitor for and avoid stakeholder fatigue over the course of lengthy trials” (World Health Organization, 2021a).

In fact, the National Academies of Sciences (National Academies of Science, Engineering and Medicine, 2016) recognised that “a universal method [for effective community engagement] that can be applied to the area of gene drives, or any other emerging technologies is unlikely”, and that as a result, researchers would need to be flexible in adapting existing models and approaches to their specific work and context. There is no definitive “how to” guide for engagement for gene drive research that can be the benchmark for evaluating current practices. However, principles and practices established in other fields as well as in the guidance frameworks cited above, offer a broad framework to support, inspire and guide researchers and stakeholder engagement practitioners (UNICEF, 2020; World Health Organization, 2020; World Health Organization, 2021a).

The challenges of community and stakeholder engagement are well documented in the literature and usually commonly agreed upon by practitioners (Gbadegesin and Wendler, 2006; Tindana et al., 2011; Reynolds and Sariola, 2018; Adhikari et al., 2020). Those challenges might have different weights or importance according to the context–for instance the literacy level–but they tend to be quite universal as the engagement around COVID-19 demonstrated. They range from ethical considerations regarding inclusiveness to practical challenges for communicating the complex scientific concepts underpinning an intervention, to the tension between inclusive engagement and traditional governance structures (Akintobi et al., 2020; Loewenson et al., 2021). Researchers have proposed overall frameworks for engagement in this field, which are useful reference points for any practitioner planning or implementing an engagement strategy (Lavery et al., 2010; King et al., 2014; Thizy et al., 2019).

The question of the role of communities, or patients, in health research is familiar. Patient-centered and community participation in global health research, and increasingly in global health governance, have marked the last decades and are now predominant in the global health discourse. Historically, health initiatives were predominantly top-down, with decisions and research agendas shaped by experts and institutions. However, the recognition of the limitations of this approach, coupled with the growing emphasis on human rights and equity, has spurred a reevaluation of conventional models (Reynolds and Sariola, 2018). The emergence of patient-centered care acknowledges the significance of involving individuals in decisions about their own health, fostering a more holistic and personalized approach. The HIV/AIDS pandemic served as a pivotal moment in catalyzing this shift towards the active participation of communities and patients (George et al., 2015). Patient activists group led this work, with the slogan “Nothing for us without us”. Activists advocated for their right to be active participants in decision-making processes related to research, drug development, and healthcare delivery. Their efforts not only contributed to expedited drug approvals but also emphasized the importance of listening to the needs and experiences of those directly affected by the disease. The involvement of communities in designing and implementing interventions became a hallmark of the HIV/AIDS response, showcasing the effectiveness of a participatory approach, and inspired a similar approach for other diseases and health interventions (UNAIDS, 2011; Lavery et al., 2010; Boulanger et al., 2013).

Concurrently, community participation emphasizes the importance of engaging local communities in the design, implementation, and evaluation of health interventions, recognizing their unique contextual insights. This paradigmatic shift is rooted in a broader global health movement advocating for inclusivity, equity, and empowerment. As evident in the World Health Organization’s Framework on Integrated, People-Centered Health Services, these principles underscore the need for a collaborative and participatory approach to addressing global health challenges, emphasizing the centrality of individuals and communities in shaping health policies and research agendas (World Health Organization, 2016).

While African voices have called for more stewardship of malaria research from African stakeholders (Erondu et al., 2021), community participation has often been quite marginal (Whittaker and Smith, 2015). Target Malaria has set the co-development principle as one of its core values, intending to innovate in this field to foster meaningful community engagement (Roberts and Delphine, 2022).

**FIGURE 1:**
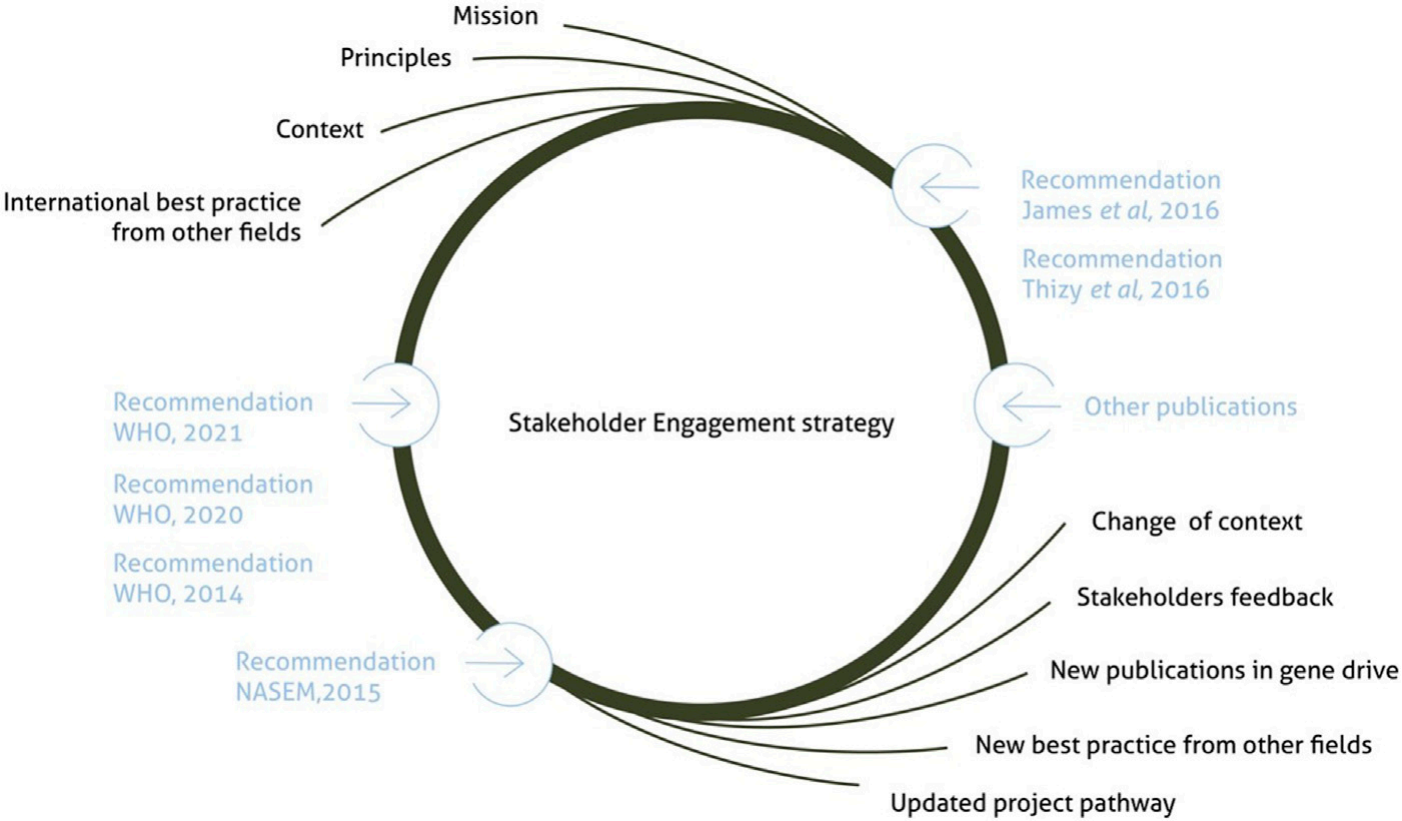
The evolution of Target Malaria’s engagement strategy: an iterative process (Pare Toe et al., 2022).

The team from Target Malaria Mali was integrating the emerging guidance as it developed, following the reflexive nature of Target Malaria’s engagement strategy approach (see Figure 1).

## Good practices and lessons learned

In reflecting upon their experiences conducting stakeholder engagement activities for the project, Target Malaria Mali staff identified three core thematic areas in which key learnings had been noted that could be useful in informing their future efforts and that of those undertaking similar work in African contexts, notably in West Africa. The first of these was lessons learned with regards to the communication of complex and novel scientific concepts; the second was lessons learned with regards to building partnerships with stakeholders, as an element of building a “co-development” approach; the third thematic area was lesson learned with regards to ending or transitioning project phases, (see Box 1 below).

BOX 1

1- Good practices and lessons learned with regards to communication of complex and novel scientific concepts
  • Adopt a multiplicity of communication strategies to meet different audiences’ preferences and to adapt to socio-cultural context
  • Use iterative processes to develop communication materials that are as effective and relevant as possible
  • Leverage different types of knowledge and foster multi-disciplinary collaborations
2- Good practices and lessons learned in developing partnerships with stakeholders
  • Involve stakeholders from the very beginning of the project and at all phases of its work.
  • Foster relationships with designated representatives of stakeholder groups who can provide key insights and local knowledge
  • Make use of several different types of in-person consultation and provide a variety of channels for stakeholder feedback
  • Provide opportunities for stakeholders to have direct and/or hands-on experience of project activities, equipment, and processes.
  • Ensure that grievance management mechanisms are in place, are contextually appropriate, and are used by local stakeholders
3- Good practices and lessons learned with regards to ending or transitioning project phases
  • Develop an explicit, comprehensive, and timely strategy for each ending or transition phase of the project
  • Develop jointly with stakeholders means of managing shared assets in a manner that is equitable and considered legitimate by local communities, and that does not preclude any future work with those communities

### 1- Good practices and lessons learned with regards to communication of complex and novel scientific concepts

#### Adopt a multiplicity of communication strategies to meet different audiences’ preferences and adapt to socio-cultural context

A key component of effective communication is engaging with stakeholders in a format that is not only intelligible to them, but that they consider relevant to their communities and interests (World Health Organization, 2017a).

The fact that communities are not only able to understand outreach activities, but that they are also motivated to take part in them (Barry et al., 2020), is foundational to meaningful and voluntary consent (Rotimi and Marshall, 2010) and more generally to meaningful engagement with all stakeholders, including at national and regional level. The challenges of achieving this in contexts where there may be large discrepancies in levels of literacy and scientific knowledge have been well documented (Crigger, 2001; Staunton et al., 2018).

This is true in all stakeholder engagement activities and not unique to Target Malaria, but the novelty of the technology made this particularly challenging. The tool proposed by Target Malaria could not be easily compared to other research projects stakeholders may have been exposed to, so it required building stakeholder understanding from the ground up.

Target Malaria Mali started engagement early, well in advance of activities that could lead to concerns among stakeholders or that could be perceived as having the potential to impact them, so that stakeholders would have sufficient time to understand this work. For example, at the national level, engagement of national stakeholders such as the relevant ministries, national malaria control programme and others started in 2014, well in advance of the project seeking permission to import non-gene drive genetically modified mosquitoes for work in containment. At the start, engagement was at a low level of intensity, but this enabled a progressive dialogue to be established so that national stakeholders were aware of the project even if there was no particular approval or permission sought from them. Similarly, the community living around MRTC was engaged from 2015, throughout the process of upgrading the insectary, getting the new facilities inspected and the concomitant research protocols approved, until the non-gene drive genetically modified male sterile mosquitoes were finally imported in 2019.

To engage the academic and research community on campus, the team also held “café scientifique” (scientific “cafés”), as a space where scientists could meet to discuss issues related to the project’s research. Overtime, some of the scientific cafés were also open to members of the public who were invited to take part in the exchanges. The informal setting of these “cafes” (all participants are sitting in a circle rather than the more usual theatre setup of academic presentations) helped foster an open dialogue between the different participants (Pare Toe et al., 2022).

Target Malaria Mali’s efforts to tailor their engagement to local contexts were extensive and multi-pronged (Wanyama et al., 2021). Initially, Target Malaria’s engagement at the community level relied primarily on verbal communication, with the support of visual aids such as large-scale printed visuals or posters in interactions, while presentations, briefing notes or brochures were mostly targeted towards national and regional stakeholders. Over time, the team diversified its approach in order to ensure the different learning styles of audiences could be taken into account, and to mitigate the risk of audience ‘fatigue’ due to excessive information sharing given the long timeframe for the project’s activities. This aimed to improve understanding of the information shared but also engagement with the team.

For example, the team developed traditional theatre performances making use of local musical traditions to engage local stakeholders. Local communities played a key role in identifying and suggesting local artists who could help communicate the intentions and details of the project through narratives adapted to local tastes and levels of familiarity with the project.

**FIGURE 2:**
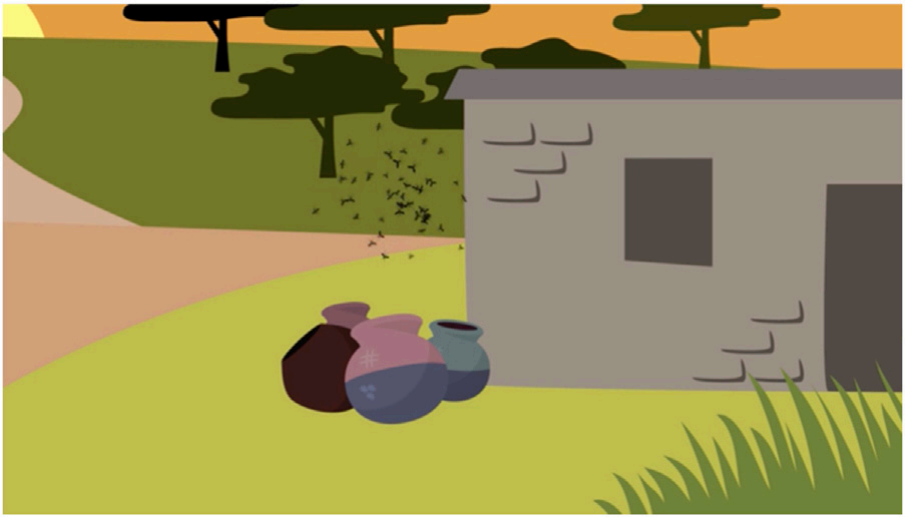
Still of a video explaining the swarm sampling activity, used to explain entomological activities to local residents before askign for their permission to carry out the activity (copyright @Target Malaria).

The team has also used videos to support discussions around complex topics. For the communities where the project conducted entomology studies, Target Malaria developed animated videos to help make the entomology activities easier to understand and to have a more varied set of tools to engage with the local community (see a still from a video in Figure 2). As the team working in villages is required to receive either individual household consent (for any indoor activity in a house, such as the use of spraying for indoor collections) or community agreement (for entomology activities taking place in the common outdoor spaces of a community, such as swarming collections), each method has to first be understood before consent or agreement can be sought for the activities to take place. For example, there can be a confusion between the Indoor Residual Spraying (IRS) activity carried out as a vector control tool as part of the National Malaria Programme (World Health Organization, 2015; Tangena et al., 2020) and the Insecticides Spray Catch implemented by the project to collect mosquitoes (Target Malaria, 2020). There is an ethical imperative in ensuring that residents understand that they are consenting to a research activity that does not provide protection from malaria.

The animated videos had voiceovers in local languages to introduce the project, introduce the overall entomology protocol, and explain two key methods, insecticide spray catch and swarm sampling. The videos responded to a need from the stakeholder engagement team to ensure activities would be explained to each resident in a consistent way, and consent recorded systematically. It also met the local residents’ preference for visual communications, while overcoming challenges linked to literacy. The videos were put on tablets that could be used during stakeholder engagement activities and complement one on one meetings and village-wide meetings. They were additional to posters, large A5 printed ‘storyboard’ illustrations, and written flyers that the project also used in meetings.

Developing the animated entomology videos across all the Target Malaria teams (in Mali, Burkina Faso, and Uganda) was a challenging process. Visuals needed to be sufficiently relatable across different cultural and geographical contexts that residents would identify with the videos, and linguistic differences made adapting voiceovers in the different languages to the same visuals complex. The experience with the videos yielded several lessons learned. Overall, the Target Malaria Mali team’s perception was that the videos were useful as residents seemed to remember more easily information that they could see, and the videos made the process less tedious. As the formal consent forms are lengthy, walking the residents through them verbally takes time and attention, the videos helped complement the forms. They were however only used for visits to individual households, not for village-wide information sessions. In addition, in Mali, when the videos were introduced, the team had moved to annual consent or community agreement for the activities, following several years of getting permission upon every visit, in response to stakeholder fatigue with the repetitiveness of the process. This reduced the frequency at which videos were used. The use of videos was interrupted when field entomology activities were paused due to the COVID-19 pandemic, resulting in the team only using the videos for a year. As videos represent a significant investment of resources to develop, weighing how much they will be used *versus* the time and cost investment put into them is important, even if they are objectively a more engaging tool.

**FIGURE 3:**
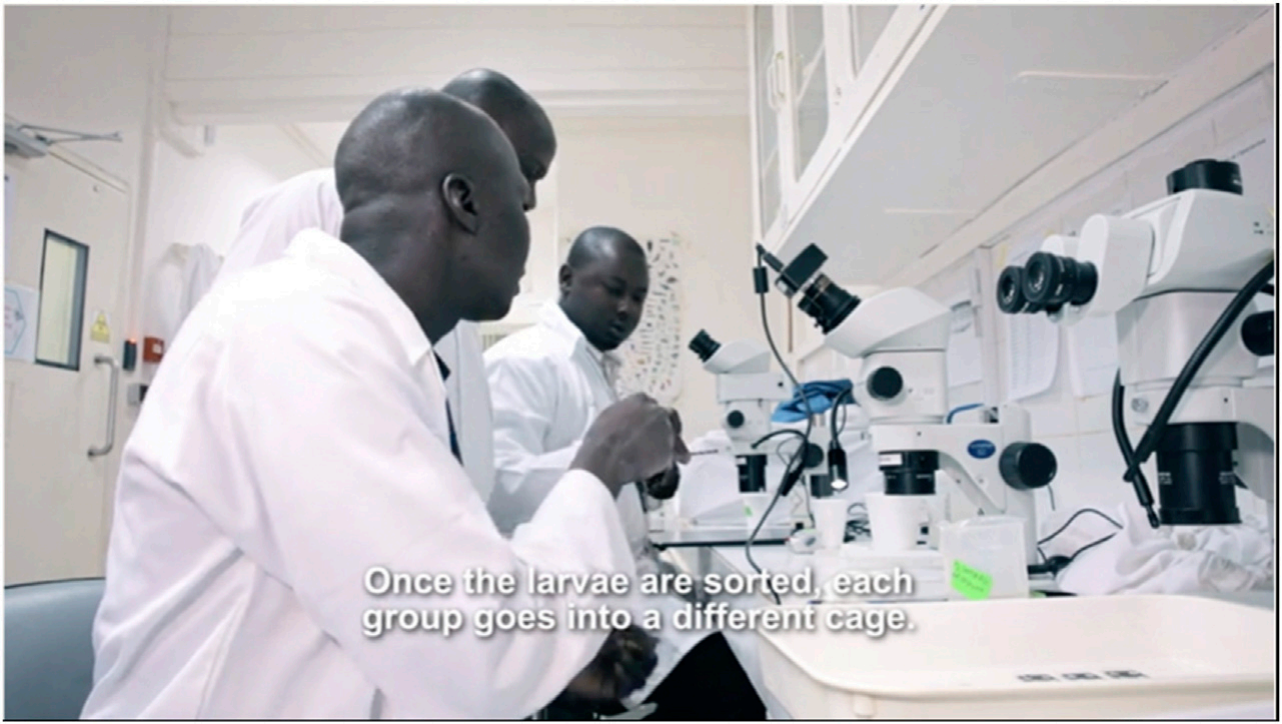
Still from the video tour of the Target Malaria Mali insectary and laboratory at the University of Sciences, Techniques and Technologies of Bamako.

Different from the entomology animated videos, the project also developed video tours of their insectaries, which have been used consistently over the past 5 years (see Figure 3). In-person visits to the insectary were a useful tool to demonstrate and explain to various stakeholders the research taking place. However, managing frequent visits could be disruptive to the team’s work. The insectary at USTTB is relatively small and visitors moving through the facility easily interrupt research activities. In addition, the team had to prepare for higher levels of restrictions on access to the insectary once it progressed with its research, notably planning ahead for research on genetically modified mosquitoes. Complying with containment and biosafety measures added to the complexity of arranging and managing visits from external visitors. To address this dilemma, the team developed filmed versions of the insectary visits (Target Malaria, 2018). These videos were made available to the public at large and have been used primarily in meetings with officials at the regional and national level. While the videos were intended to address a challenge faced by the Target Malaria Mali team (and other country teams), one of the outcomes is that in fact they have been most useful for stakeholders outside of the country, and in particular with media.

#### Use iterative processes to develop communication materials that are as effective and relevant as possible

Early-stage engagement with many communities involved assessing their knowledge and understanding of malaria as a first step, and thereafter building a series of conversations around key concepts (malaria, genetics, inheritance, the role of mosquitoes, etc.) so that, over time, stakeholders could meaningfully engage in conversations about the project’s work. It also gave them the time to seek additional information if they wished to add to what the project was providing.

**FIGURE 4:**
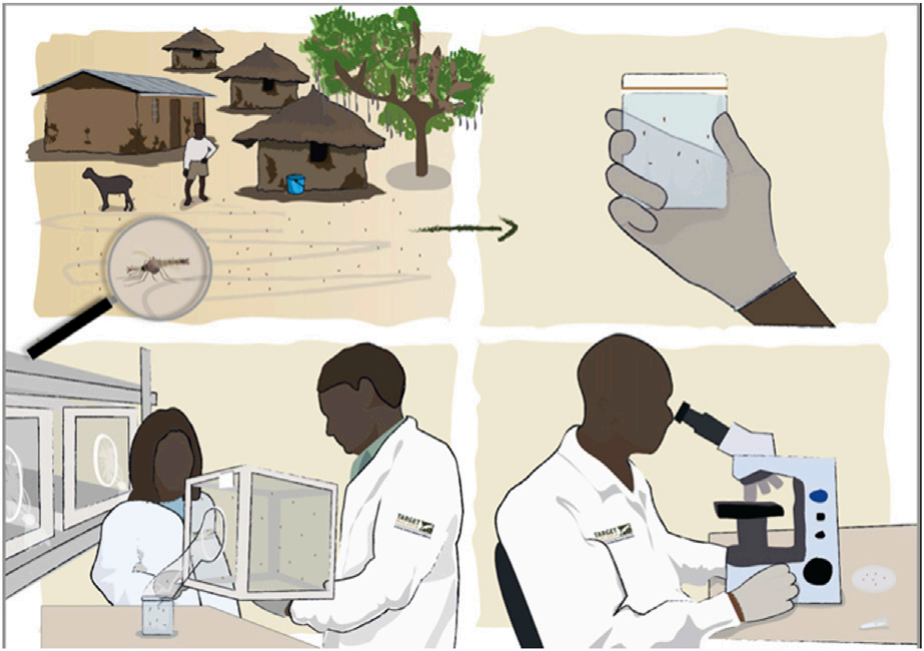
Example of a visual developed to support engagement with stakeholders at community level, describing the process of collecting mosquitoes from local sites and bringing them to the lab to be studied (copyright @Target Malaria).

One tool developed at the project level to support this “step-by-step” approach was a set of hard-copy visual aids that were printed in large format and used to reinforce stakeholder understanding (see Figure 4). These enabled the team to combine verbal and visual engagement, which was useful when introducing key concepts, such as genetic inheritance. The set aimed to offer a visually realistic representation of concepts and activities, while being appealing and easy to grasp. One of the challenges in developing this tool was deciding the appropriate level of detail to depict so that the visuals would be useful without being burdensome. To overcome this challenge, the visuals were developed as “chapters” to be used by the teams in sequence, but with the ability to only use some visuals or all depending on audience feedback and prior engagement. The visuals are each printed on a standalone A1 size sheet and can be used independently. Once these visuals were finalised, workshops were organised with the various teams to elaborate how they would use these visuals in their specific contexts, using the linguistic work done to further tailor the message to the specific audiences. In Mali, the Target Malaria Mali’s engagement team started using these visuals around the insectary for the engagement about the project and ahead of the application for the importation of the first non-gene drive strain of genetically modified mosquitoes. During these sessions, it appeared that the visuals were a highly appreciated tool, demonstrated by the requests from some key community members (such as the schoolteachers) to be given this tool and trained.

In addition to being tailored to local contexts, many of the new materials (such as the Bamanankan glossaries mentioned below) were created through iterative and stepwise processes in order to allow for multiple rounds of feedback, seeking stakeholders’ opinions and inputs on the material and refining them to ensure that they were supporting the stakeholders’ understanding.

#### Leverage different types of knowledge and foster multi-disciplinary collaborations

Target Malaria Mali’s stakeholder engagement activities were the product of a collaboration between many different subject matter experts within Target Malaria. They drew on inputs not just from entomological researchers and stakeholder engagement experts, but also from those working in public relations, regulatory affairs, and genomics, among other fields.

For example, the visuals used to describe the project, as well as the videos used to explain specific entomological activities, required extensive internal collaboration between the entomology, laboratory, stakeholder engagement and communication teams. They were first developed by Target Malaria’s global communications team, who then worked with the team out of Mali to translate them into local languages and ensure that the narratives developed therein were culturally appropriate.

In addition, the Target Malaria Mali team made use of third-party experts from beyond the remit of the project itself (for example, linguists from the national language institute). Finally, a third strand of knowledge informing their communication efforts was that of the stakeholders themselves. Inputs from stakeholders on the concepts and terminology that was being used to communicate with them were vital in refining them until fit for purpose.

The example of the French/Bamanankan language glossary helps highlight the need for collaborative engagement with multiple stakeholders to support stakeholder engagement (Wanyama et al., 2021). Target Malaria aims to ensure a constructive dialogue with its stakeholders. While at the national level most engagement activities were carried out in French, engagement with local communities requires communication in different languages. The Target Malaria team worked with local communities and linguistics experts to develop a language glossary that established common understanding around key terms and concepts that were core to discussions about the project.

The first step in this process was a 4-day workshop held at the Malian Academy of Languages (AMALAN), co-organized by Target Malaria Mali and the AMALAN. The main objective of this workshop was to offer translations for key concepts and genetic expressions from French into the Bamanan national language which is referred to as “Bamanankan”. Bamanankan is widely used in Mali and in other countries in West Africa, making communication in this language a priority for effective engagement. The workshop started with sessions during which the concept was explained and discussed until participants had a common understanding of the ideas that needed to be conveyed. Once this was established, participants then turned to the question of translation, discussing which term to use and in particular which ones would be most accessible to participants in future engagement with the project.

Following this theoretical phase, the Target Malaria Mali team worked with local residents to get feedback and validate the terms chosen. In the two villages where the project was still active, ten people, all neo-literate in Bamanankan, were chosen by the community to participate in “proof-of-concept” sessions. Concepts were first explained in Bamanankan by the Mali principal investigator of the Target Malaria project, then explained a second time in Bamanankan by the AMALAN representative to offer a second perspective and nuances. Participants were asked to share their understanding of the concepts and to say which words they would use to name those concepts. The team then shared the terms that had been suggested in the first workshop with the AMALAN. By the end of the 2-day session, the recommendations and suggestions made by the communities were recorded and incorporated into a final version of the glossary. The glossary, entitled “Recherche et linguistique au Mali: essai de transcription de notions génétiques du français en bamanankan” is now available to all researchers and others working in Mali through MRTC, AMALAN and some other research structures.

While this effort was very successful and delivered a tool that has been used extensively by the Target Malaria team, it was not without challenges. There are few scientific researchers who have experience working and communicating in the national languages outside of French and English, meaning there was not a strong baseline or a lot of prior experience to draw upon. The “language of science” in academia is predominantly French and English, as these are the languages in which researchers communicate to funders, in which they publish, and in which they were taught. Even for those who do have knowledge of the local languages, discussing complex scientific topics was not common and seeking to develop translation is not a standard practice. On the part of the communities, residents are also not used to engaging in discussions on these topics. Doing so required a significant number of prior exchanges to build understanding before agreed translations could be found. The clear lesson learned from this experience was that having a glossary (and the process to build common understanding to then develop the glossary) was immensely useful and improved communication between the team and the local community. However, this is a complex and lengthy process that should be factored in as an early step in research projects’ activity plans to help build stronger communication and avoid early misunderstandings. One of the outcomes of the experience has also been that all new stakeholder engagement staff for Target Malaria Mali were required to take a course in Bamanankan to improve their fluency and ensure they could easily speak with local communities.

### 2- Good practices and lessons learned developing partnerships with stakeholders

#### Involve stakeholders from the very beginning of the project and at all phases of its work

The successful implementation of a collaborative approach requires that stakeholders most affected by the research project play a meaningful role in decision-making on how that project’s research activities are conducted (Adhikari et al., 2017). This means that they must be involved from the early stages of project planning and implementation, and that this involvement must continue in every stage thereafter (National Academies of Science, Engineering and Medicine, 2016; World Health Organization, 2021a). Target Malaria Mali’s early engagement with local stakeholders before any field research took place (in the case of the villages) and before work with genetically modified mosquitoes began in the laboratory (in the case of the communities around the insectary) helped foster a relationship of mutual trust and respect between researchers and stakeholders that enabled effective cooperation throughout the lifetime of the project (Pare Toe et al., 2022). The initial engagement was diverse, encompassing various stages such as information dissemination, consultation, and feedback. The information activities were designed to render information accessible to stakeholders, ensuring it was presented in a manner that is not only comprehensible but also holds significance for them. Engaging in consultation meant actively interacting with communities to collect their input, feedback, and perspectives, ensuring that their interests and concerns shaped the decision-making process. These consultations varied in formality, ranging from more structured sessions to informal exchanges. It is important to note that seeking individual consent or community acceptance represents just one facet of the diverse range of consultations held. Finally, the feedback was a phase in which the project would come back to the community or stakeholders to share the knowledge that had been built during the research process, for instance sharing the results of the entomological collections with communities where collections had taken place.

In the first few years of the project, the team in Mali worked in four villages, with the community living around the campus where the insectary is located, and with regional and national stakeholders. During these years, the team carried out monthly visits to the villages, for a total of about 10 days a month. In this early phase, the engagement was meant to not only introduce the project, but also support the entomology team undertaking monthly entomology collections. Overtime, the team moved to seeking yearly rather than monthly agreement for entomology activities.

The move to yearly agreement for entomology activities is illustrative of how residents’ feedback shaped Target Malaria’s activities. Initially, seeking permission every month for the activities seemed necessary and best-practice. It was meant to enable residents to quickly signal a change of mind about the activities and withdraw permission, while enabling the team to have frequent interactions to gage issues and concerns. In order to ensure traceability, the team was recording the permissions in writing and performing both community-wide sessions and visits to individual households.

Over time, however, residents expressed fatigue with the frequency of the requests for permission. The entomology activities were consistent enough that, once familiar, residents conveyed that they were sufficiently aware of the work without needing monthly reminders. This was testament to the relationship built between the Target Malaria team and the residents, but also demonstrates that what is best practice needs to evolve over time to take into account residents’ needs and preferences. This was also grounded in the socio-cultural context of the villages where the project worked. While externally the notion of asking for written permission each time seemed to be best to enable residents to withdraw or express disagreement and to offer transparency, in the local context it went against the accepted practice that predominantly gives social value to verbal agreement. Repeatedly seeking written permission could at times be interpreted as a lack of trust or respect for the residents by seeming to imply that the previous agreement was not trusted.

Taking these elements of feedback into account, the project moved to yearly agreements, after approval of this change by the MRTC research ethics committee, while continuing to carry other activities (like feedback sessions on findings) in between. In a research project that will necessarily carry on over a long period, resident feedback on preferred procedures is also an important form of co-development of the research pathway.

#### Foster relationships with designated representatives of stakeholder groups who can provide key insights and local knowledge

In the early stages of its work, the project made contact with local volunteer “guides”. Guides were members of the community that were selected by the community to work with the project. Target Malaria outlined some key criteria and the residents put forward volunteers. These guides were key to the success and co-development of the research on several fronts. As trusted members of the communities, they facilitated introductions between the research team and its other members, serving as a vital intermediary and strengthening trust between all parties. Their geographical knowledge was also essential to identifying mosquito breeding sites around communities, an important initial input supporting the early stages of entomological research. The guides were involved from the very beginning of this research, which was important to avoid mistakes and build trust, and they were vital sources of informal feedback and perspectives on community dynamics and potential concerns that may be developing. These findings are consistent with the experience of other research projects, such as those with Mass Drug Administration in Laos (Adhikari et al., 2017) or in West Africa with Insecticide Treated Nets (World Health Organization, 2008) and in keeping with numerous recent studies on best practices in global health (Adhikari, Pell, and Cheah, 2020; Hickey et al., 2022).

In addition to the guides, the project also established permanent representation in the villages. This mechanism was established to ensure continuity in the presence of the project when the frequency of team visits was reduced, but also meant to ensure that residents would have someone locally available and who knew the community well that they could direct questions to. The “project representatives” were from the community and each was responsible for two villages. They were selected based on baselines criteria through a committee made of community residents and project members. Their role was to relay information and questions back and forth between team visits, and to have informal interactions with residents to allow conversations to develop organically rather than only in the structured setting of a community meeting or household visit.

The project also built or repurposed houses in the two main villages to serve as the ‘project house’. While this served to house team members when doing field work, it also had the purpose of serving as a base for meetings and a place where information could always be available. The project houses were equipped to meet the standards of an office and were a place where the residents could go to seek out information about the project. The project credits this initiative with significantly helping create trust between the residents and the project, and the consistent openness of the local communities to project activities.

To ensure transparency of activities but also to increase stakeholder engagement in the project, the project also created in collaboration with local residents monitoring committees that would provide oversight to the implementation of activities. These committees were composed of members of the community who would participate in some of the entomology activities and also monitor that activities agreed to were implemented appropriately. At the insectary level, a consultative group was put in place to help the project explain the planned importation of the genetically modified mosquitoes and to facilitate consultation with the community around the insectary prior to seeking their approval. This group was also made of community members with the participation of project members.

Fostering relationships was also important at the national level. Faced with frequent changes in government officials which impeded awareness of the project, the team devised a system of focal points among key ministries’ civil servants. These were individuals who could be informed regularly and who were then able to transmit information about the project to colleagues in their institution. They were also contact points for the team when they needed information about procedures or updates. These relationships were formally established: once possible focal points were identified, and if they agreed to take on this role, they would first participate in a workshop and then receive quarterly information via the rectorate of USTTB where the Target Malaria Mali project was hosted. In addition, the stakeholder engagement manager would regularly interact with the focal points to share additional information and receive feedback. This system, established in 2019, enabled the team to weather several changes in government leadership and offered easier contacts throughout the pandemic in 2020 and 2021. It is a system that can be replicated and scaled up and which offers teams with limited resources an effective way to manage relationships with key government institutions.

#### Make use of several different types of in-person consultation and provide a variety of channels for stakeholder feedback (continuous and annual, including different formats)

The use of diverse channels for information sharing helped the project to reach out to, and hear from, a wide range of stakeholders. For example, those who might not have been interested in attending “town hall” meetings or reading written pamphlets or explanations might still be drawn to attending the theatre performances. And those who may not have been comfortable speaking up in front of community meetings may be more willing to share their views in smaller groups during household visits. Similarly, the use of different mediums for information sharing helped the project to live up to its principle of inclusivity, as relatively marginalised stakeholder groups (such as women and youth) are not able to participate on equal terms in all information sharing venues.

For example, the team was well aware that women participating in wider meetings may not be considered decision-makers and that they may not express their opinions freely to avoid contradicting men in the group. The team noticed in early community meetings that women never took the floor or raised questions. As it is common for women to gather separately in the community, the Target Malaria Mali team built on this practice and created distinct women groups to hold discussions about the project. The women’s groups were called the “tontine” groups and the project was invited by women to come to join them in the meetings. During those “tontines”, women raised questions and participated, showing that adapting the setting for the project was a more effective avenue to engage women of the community.

Similarly, for youth in urban settings around the Target Malaria Mali insectary, pre-existing social groups called “grins” could be leveraged to engage younger people in the community without artificially creating new groups. A “grin” is a social space for reunions and talks for people in the neighborhood rather than a group with a set membership - as such the “group” is the participants that come to the designated place at the given time, making it variable in its composition. “Grins” stem from the social practice of drinking tea in an informal group, often gathered outside of homes (in the street), originally primarily composed of men gathered around a grin (Bondaz, 2013).

There can be many “grins” in a single community with different people gravitating towards different locations. During gathering in the “grins”, social issues such as health, education, sanitation as well as political news are discussed. The groups may bring together participants around shared backgrounds or characteristics, such as studying in the same faculty, but are not highly homogenous. A “grin” can bring together community member of different ethnicities, in different professions, unemployed or studying. Youth in a community may gravitate towards selected grins, offering an entry point to engage with them in a pre-existing space.

“Grin” locations were identified by the engagement team who walked around a certain neighbourhood. Identifying one ‘grin’ led to another, highlighting the informal and fluid nature of these groups. The lessons learned from this experience was that engaging directly with the “grins” was an effective way to convey messages and receive more relevant questions directly from youth in a setting in which they felt comfortable and in charge. It enabled wide dissemination of information as “grin” participants shared what they had learned with others in the community, and it offered a highly participatory setting for engagement. However, it was relatively more limited in the ability to provide long term engagement to a consistent group of people and often required repeating and revisiting prior discussions as new participants joined the group and all members not being always present at the same time.

The project’s decision to also add household visits to the initial plan for community-wide sessions and focus groups was similarly an effort to ensure different people in the community would be engaged, across social structures and including women who may not attend village meetings. It was also a way to accommodate residents’ schedules and constraints, in particular during periods of harvest or planting when most did not have time to attend community or group meetings. Household visits required additional time and effort from the project team since it added to the schedule of activities, but they found that it allowed them to build more direct relationships with residents and reach a more varied cross-section of people in the community, particularly as they would meet household members who may not participate in public meetings.

#### Provide opportunities for stakeholders to have direct and/or hands-on experience of project activities, equipment, and processes

Related to the above lesson-learned, the project found that certain forms of engagement and information sharing were more likely than others not only to elicit the interest of stakeholders, but also to meaningfully improve their understanding of the project’s work. These tended to be forms of engagement in which stakeholders were given direct experience of the methods, tools, and spaces with which research was conducted. The importance of stakeholders directly “experiencing” and participating in the research has been noted as an important factor in building trust and in supporting the co-production of knowledge. For example, Tembo et al. note that “to deal with the power imbalance between researchers and communities, and within research collaborations, it is important to include experiential knowledge and participatory methodologies” (Tembo et al., 2021).

For communities living near the insectary lab, the project organised guided tours of the laboratory for stakeholders. This helped to dispel misconceptions, demystify the work of the project, and give stakeholders a sense of what day-to-day research entails. They were able to experience for themselves that nothing overtly mysterious or sinister was taking place behind closed doors, and that the project was committed to transparency and making its work accessible. The stakeholder engagement team records interactions in a database and comments received following the visits indicated that the participants’ reaction to the visits were positive. It was notable that many noted that it was their first visit to a research laboratory and considered this opportunity to physically see the research as an important element of transparency.

This principle was also true of engagement activities away from the lab, for example, in community meetings near areas where field entomology activities were carried out. Stakeholders expressed a greater level of enthusiasm and understanding when project members brought examples of the type of equipment they would be using for these studies, and provided demonstrations of how they were used and allowed those present to handle them. Local community members also volunteered to take part in several different kinds of entomological collection activities. Volunteers were given training to allow them to identify male and female mosquitoes and differentiate between the genera of mosquitoes present in the area, such as *Anopheles, Aedes,* and *Culex*. This helped to build understanding among community members and to allow them to feel invested and knowledgeable as partners in the research.

#### Ensure that grievance management mechanisms are in place, are contextually appropriate, and are used by local stakeholders

A commitment to accountability is not meaningful unless stakeholders have an accessible, responsive, and easy-to-use mechanism to register any complaints or other grievances they may have with the project (UNICEF, 2020). A grievance management committee was established for each community which was near project activities, composed in equal parts of project members and representatives of those communities, which the communities themselves chose. This committee was responsible for analysing grievances submitted and determining the best course of action to address them and informing project management if applicable. Grievances could be communicated to the members of the Committee in many forms (for example, verbally, via WhatsApp, or in writing). A register of all grievances received and the actions taken to address them was maintained.

The project found, however, that no grievances were received via this channel. In response, an internal evaluation was conducted in consultation with stakeholders to determine whether the mechanism was fit-for-purpose. The evaluation showed that residents knew who the members of the committees were and could cite at least two ways in which complaints could be made (in person or by phone) and did not feel that there were impediments to meeting with the committee members (whether due to lack of trust, access, or knowledge). At the same time, respondents also did not cite the mechanism as their first port of call to register complaints and overall did not indicate feeling the need to register complaints.

While the evaluation showed room for improvement in uptake, it did not entirely indicate why no grievances were received through the mechanism. Reflecting on the experience, it is likely that several factors came into play. The evaluation and other feedback sessions with stakeholders indicated that, overall, residents felt they knew the team members well and estimated that the project operated with transparency. Residents noted that project members were readily available to answer questions and hear out concerns. This may have contributed to residents not feeling aggrieved and, when they had concerns, preferring to raise them in conversations or exchanges with team members in an informal way, rather than raising them to the perceived level of importance of a grievance. In addition, the establishment of project representatives and village houses in each site might have constituted an easier path to answering residents’ questions rather than going through the grievance mechanism. Finally, socio-cultural factors, such as a preference for oral communication, the impact of social hierarchies which may make some residents (and in particular members of groups with less social standing, like women) reluctant to approach committee members or voice grievances.

The lack of formal grievances does not preclude that some residents would have had at times complaints, which would seem inevitable during a 10-year collaboration. However, it does indicate that a combination of factors must be taken into account to explain why the grievance mechanism, while known and acknowledged, was largely unused. This experience shows that while grievance mechanisms are seen as a well-established and vetted best practice in this field, many factors come into play in determining what is the most appropriate way to enable grievances to be expressed and addressed. This can be challenging to justify to external audiences who have certain expectations of set practices, but it highlights the fact that in some cases there may be tensions between the recommendations of accepted guidelines on stakeholder engagement and the particularities of stakeholder preferences and needs in a given context. Research projects must therefore be able to adapt and innovate with regards to grievance management based on those preferences and needs.

### 3- Good practices and lessons learned with regards to ending or transitioning project phases

#### Develop an explicit, comprehensive, and timely strategy for each ending or transition phase of the project

The process of ending or transitioning a project phase can be a delicate and challenging one. Yet there is little guidance available about to manage the “exit” or end of stakeholder engagement in a global health research project. Those that address this topic, such as the guidance from the Nuffield Council of Bioethics, do so mostly in the context of health research involving individuals, and focus on the question of continued care and treatment and what is owed to participants in human subject studies (Nuffield Council on Bioethics, 2005). Most research protocol require an “end of study” procedure to be specified, but in multi-year projects such as Target Malaria, where in effect series of studies occur, the “end of study” procedure may not be the same as what is needed for the end of the overall project. The team had to consider how to end its work in a way that would maintain the trust and good relations that had been built with the community. This required offering transparency, providing clarity to stakeholders and managing their expectations.

This is particularly challenging for a project that has emphasised co-development and stakeholder participation because the decision to exit sites was not the result of a breakdown in relations with the local community, but instead due to changing needs for the scientific progress of the overall project. As a result, it is a form of unilateral decision on the part of the project, at odds with its preferred way of functioning. Exits run the risk of leaving the local community feeling disempowered.

Acknowledging the risk and challenge that this change in dynamics created, the team sought to mitigate this situation by being honest and transparent about the reasons for the exit and by taking time to process the exit with the community so it would not be abrupt. For example, meetings were held in the community with each stakeholder group, as well as an “open” community-wide meeting to first inform the groups. These were followed up by meetings where stakeholders could share their views and questions, having had time to process the information. Finally, a formal closing meeting was held with all leadership and the community to formally close the collaboration. The complete process from initial to final meetings was conducted over a period of 6 months and involved twenty individual meeting sessions for each community.

The effectiveness of Target Malaria Mali’s approach is exemplified in the feedback they received from stakeholders. In the database of feedback, a participant who took part in the exit process is on record as having said:

“We appreciate Target Malaria's approach. This practice, of informing us of the end of the activities of the Target Malaria project in Mali, shows that the project respects the principles of transparency and accountability. Approaching us and informing us of the end of the project activities in Mali reiterates the appreciation of the good collaboration with the stakeholders. We testify that we have learned enough about mosquitoes and the various diseases transmitted by mosquitoes during these years of collaboration with you. We also understand that a project has a lifespan, sooner or later it will end one day”.

#### Develop jointly with stakeholders means of managing shared assets in a manner that is equitable and considered legitimate by local communities, and that does not preclude any future work with those communities

As described earlier, as part of several years of work in local communities, the project in many cases helped develop tangible assets held jointly with communities to facilitate their research activities, such as the “project house” and the weather stations.

When Target Malaria Mali ended its work, the team had to consider how to hand off these material assets to the community. The project house had been built on communal land given by the community, represented by the village chief and his advisers. The project was keen that the transfer should not generate conflict or perceptions of bias in the community given the fact that the house was built on property that was originally communal land, and so needed a transparent process for transfer. The team held public meetings involving the various stakeholders in the community, including the traditional authorities, and then handed over the keys to the project house to the village council in a public meeting. The objective of holding the meetings to discuss the handover and to have a public ceremony was to ensure all the stakeholders were aware of the agreement reached and to avoid misunderstandings. It is also hoped that if there were disagreements in the future over the use of the house, community members could refer back to the meetings and the public ceremony to recall the intention to keep the house as a communal space.

While this does not guarantee that in the future no one would seek to claim the house, it was important for the project to ensure the physical legacy of its time in the village continued to benefit the community.

The weather stations contributed to the characterisation of malaria vectors and seasonal variation by collecting data such as rainfall, temperature, and wind direction. That data was shared with the residents as part of feedback sessions. Beyond the value of the general knowledge sharing, residents also used the data to inform their activities, in particular agricultural activities. The original stations required a high level of maintenance and technical know-how. So, the team replaced these with simpler versions that the community could use with less maintenance before handing over the stations to the community for their use.

Finally, the Target Malaria Mali team and the Target Malaria project agreed to continue to check in with local stakeholders for 3 months after the exit was completed, to ensure that if concerns or complaints arose after the formal end of project, these could still be captured and addressed. After the 3 months no concerns or complaints were recorded.

## Conclusion

Ten years of continuous stakeholder engagement from the community to national level offer valuable ground for reflection for researchers involved in developing novel tools for vector control in West Africa. The experience of the Target Malaria Mali team reflects the fact that established best practices for stakeholder engagement are valid and continue to be relevant, even when working with a novel technology such as gene drive, while also pointing out some useful lessons learned about how specific challenges can be met.

The duration of the project and the novelty and complexity of the concepts involved in the research were two key factors that influenced how the project approached stakeholder engagement and shaped the type of activities undertaken. Managing stakeholder expectations over a long period of time, while also managing fatigue and turnover are not unique to Target Malaria, but the 10-year framework is longer than is usual for many projects. In addition, the topic (genetic approaches for vector control) was truly new to stakeholders, not only at the village level, but also to most at the national level. Given the dramatic negative impact that malaria has on people in Mali, finding the right way to engage stakeholders in the project as an endeavour that could possibly over time alleviate a great public health burden, while also avoiding misunderstanding and unrealistic expectations, was challenging.

Ultimately, Target Malaria Mali decided to conclude its work and refocus towards building on its core competencies by creating a center of excellence in molecular engineering. This represents a success, but also a change of direction that directly affected the stakeholders with which the project worked most closely, and with whom engagement had been amongst the most active. Closing out the Target Malaria Mali project needed to be done thoughtfully and in a way that was truthful to the approach the project had taken so far. Many research projects come to an end, but the duration of the project and the intensity of the engagement in prior years mean that the end of the project was very visible to many stakeholders.

In this paper, we have highlighted a series of lessons learned and good practices that others working in similar fields may find useful and which can contribute to the growing body of literature informing the conduct of responsible gene drive research.
